# A descriptive study of blood pressure in people with self-reported substance use seeking health care in region F, Johannesburg, South Africa

**DOI:** 10.1016/j.pmedr.2025.102977

**Published:** 2025-01-17

**Authors:** K.E. Oladimeji, S. Gumede, A. Nyatela, S. Nonyukela, R. Mohale, S.T. Lalla-Edward, D. Dwarka

**Affiliations:** Ezintsha, University of the Witwatersrand, Faculty of Health Sciences, Johannesburg, South Africa

**Keywords:** Elevated blood pressure, Hypertension, Cardiovascular diseases, Substance use, iHEART-SA

## Abstract

**Background:**

Elevated blood pressure is a major risk factor for cardiovascular diseases like hypertension and leading cause of death in sub-Saharan Africa. Substance use such as alcohol, tobacco and drugs, is one of various factors attributable to elevated blood pressure This study describes the prevalence of elevated blood pressure and associated factors among individuals who self-reported to substance use.

**Methods:**

This descriptive cross-sectional study involved use of secondary data from the Integrating HIV and Heart Health in South Africa study collected between August 2022 to May 2024. Primary outcome, elevated blood pressure was classified as systolic blood pressure ≥ 140 mmHg, and diastolic blood pressure ≥ 90 mmHg. Data was analysed using STATA version 17. Logistic regression analysis was performed to identify factors associated with elevated blood pressure.

**Results:**

Of the 2148 participants included, 57.3 % (1230/2148) were males. The participants' median age was 41 years, and 35.9 % (772/2148) had elevated blood pressure, however only 16,9 % of the participants were receiving anti-hypertensive treatment. Independent factors associated with elevated blood pressure were increasing age - 30-39 years old (adjusted odds ratio (aOR) 2.78 [95 %CI 1.42–5.41], 40–49 years old (aOR 3.81 [95 %CI 1.95–7.46]), 50+ years (aOR 5.00 [95 %CI 2.45–10.20] and having comorbidities (aOR 2.35 [95 %CI 1.31–4.22]).

**Conclusions:**

Prevalence of elevated blood pressure was high and only a few of these were on anti-hypertensive treatment. Most participants reported using alcohol, including both alcohol and tobacco concurrently. Findings highlight the need for substance use interventions as part of cardiovascular disease management strategies.

## Highlights

• Elevated blood pressure is a signal of hypertension – a risk factor for heart disease
• In 2019 hypertension climbed by 48 % in men and 34 % in women in sub-Saharan Africa
• In South Africa 30 % of hypertensive people use drugs; we report 64 % alcohol use
• In Africa there is limited evidence linking substance use and cardiovascular diseases
• We show a link between substance use and elevated blood pressure - warranting focus

## 1 Introduction

Elevated blood pressure is an indicator of hypertension and other cardiovascular diseases, which are a critical public health concern globally, and their prevalence is particularly high in Sub-Saharan Africa. (Gafane-Matemane et al., 2024; Goff et al., 2014; World Health Organization, 2023). Over the years, the definition and categories of hypertension have evolved, but it is widely agreed that persistent blood pressure readings of 140/90 mmHg or higher warrant treatment (Iqbal and Jamal, 2023). To be diagnosed as hypertensive, one must have elevated blood pressure readings taken on at least two separate occasions. The most recent report published by the World Health Organization, stresses the pressing need to address hypertension and its associated impediments, that impact more than a billion people (World Health Organization, 2023). This condition is a major risk factor for cardiovascular disease and among the leading causes of morbidity and mortality (Goff et al., 2014). South Africa has the highest prevalence of hypertension (over 77 %) compared to the rest of sub–Saharan Africa (Sharma et al., 2021). Moreover, in sub-Saharan Africa, the development of cardiovascular disease in patients is an average 15-year earlier compared to other countries globally^5^. Hypertension can be hereditary or can result from environmental influences such as bad diet, obesity, inactive regime, and substance abuse (Zhao et al., 2023; Okojie et al., 2020; Sharma et al., 2021). Substance use, including alcohol and tobacco, is known to contribute to elevated blood pressure (Zhao et al., 2023; Okojie et al., 2020; Aronow, 2017; American Heart Association, 2024), and is characteristically linked to a pattern of damaging practice of mood-altering intentions (McLellan, 2017).

Epidemiologic studies have shown that substance abuse have high prevalence rates across all age groups (Blows and Isaacs, 2022; Vasilenko et al., 2017). Substance use is a major issue in Region F, Johannesburg, reflecting broader trends seen across the city and country (Mohale and Mokwena, 2020). As reported by Mohale and Mokena (2020), 31 % of the 308 learners that they sampled, used substances. Several substances are associated with an increased risk for elevated blood pressure and cardiovascular problems. Acting as a stimulant, some substances rapidly surge blood pressure; consequently, increasing the risk for cardiac conditions (American Heart Association, 2024; Aronow, 2017), such that it may be argued as a probability of a pathophysiological relationship linking the two conditions. However, there is limited data correlating substance use and hypertension particularly in Africa. Discovering this link could fundamentally be significant as public health method directed at substance use may well reduce hypertension rates, and consequently cardiovascular complications and death. This study reports on the prevalence of elevated blood pressure and associated factors among individuals who self-reported substance use (e.g. alcohol, tobacco and other drugs such as cocaine, and dagga) while seeking healthcare in Region F, Johannesburg. By understanding the relationship between substance use and elevated blood pressure in this population, we can better inform public health strategies and interventions to manage and reduce the burden of hypertension in this community.

## 2 Methods

### 2.1 Design

We conducted a descriptive cross-sectional study involving analysis of secondary data from the Integrating HIV and Heart Health in South Africa-intervention study between August 2022 and May 2024 (reference: M211160). Integrating HIV and Heart Health in South Africa is an effectiveness-implementation study that used a randomized cluster stepped-wedge study design where primary healthcare clinics were assigned to a time at which they initiated an intervention to improve management of hypertension. This design allowed the testing of strategies to promote the integration of proven interventions in real-world practice (i.e., implementation strategies), while simultaneously assessing clinical effectiveness (i.e., patient-level outcomes) (Galaviz et al., 2024).

### 2.2 Ethical considerations

As part of the Integrating HIV and Heart Health in South Africa study, participants consented to having their data collected from their medical records and analysed. By ensuring anonymity of their data analysed, this study complied with the implementing research institution and local ethical review committee's guidelines for protecting human subjects with regards to safety, privacy and confidentiality. The current analyses are based on the initial visit of participants who opted to provide consent to be enrolled in the study and to have their records reviewed.

### 2.3 Study population and location

The Integrating HIV and Heart Health in South Africa study is conducted in 9 of 14 clinics in Region F. These clinics serve over 1 million residents and offer reproductive and sexual health care, mobile and community outreach, HIV screening and treatment, and tuberculosis screening and treatment. Between 2022 and 2024, each clinic served an average of 4500 people living with HIV and hypertensive patients (range: 2000 to 15,000) (Lalla-Edward et al., 2022).

#### 2.3.1 Sample, inclusion and exclusion criteria

**Figure 1:**
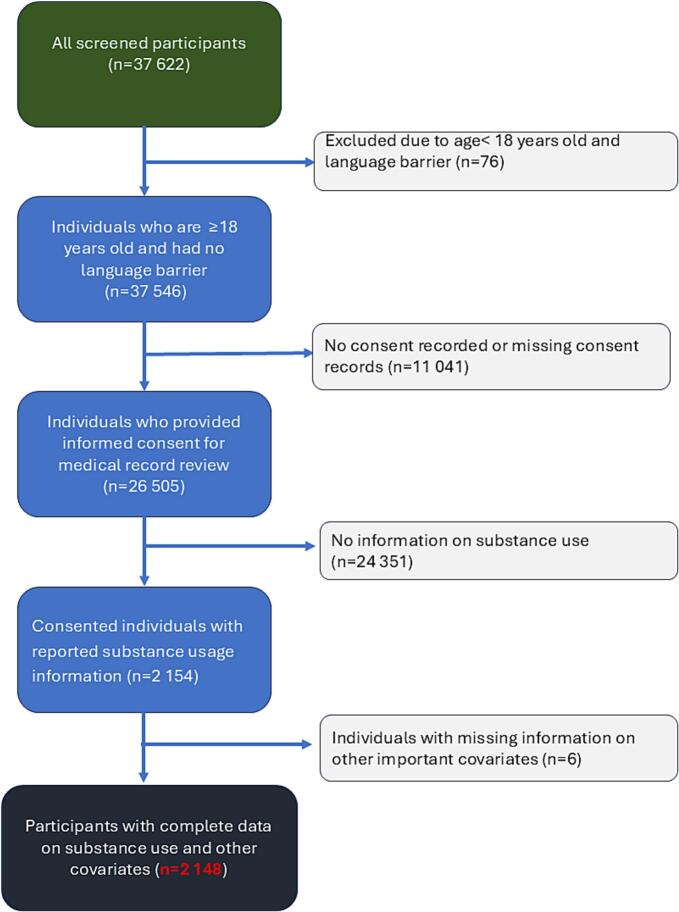
Consort diagram

The Integrating HIV and Heart Health in South Africa study included adult participants (18 years and older), living with or without HIV who consented to the medical record review. Participants were excluded from the from the study if they could not read or understand any of the languages used by the study team (English, IsiZulu, IsiXhosa). There were 26,505 eligible individuals who provided informed consent for medical record review (Fig. 1). Of this, all records of participants who consented, self-reported substance usage, and contained information of the covariates of interest (*n* = 2148) were included in our analysis (Fig. 1).

### 2.4 Data collection

Secondary data were gathered through self-reported information provided by participants during healthcare worker-administered enrolment. Additional medical details were obtained from a review of the participant's individual medical records maintained by the primary healthcare clinic. All data was captured on REDCap (a web-based data management system). The sociodemographic data consisted of the participant's sex, age, nationality, and self-reported substance use such as alcohol, tobacco, and other drugs (e.g., cocaine and dagga). The medical information gathered through record reviews included weight, height, pregnancy, HIV status; if blood pressure was measured, the measured values (systolic and diastolic blood pressure), hypertension diagnosis, date, medication, name of drug or regimen, and comorbidities.

### 2.5 Primary outcome measure

Elevated blood pressure was defined as systolic blood pressure ≥ 140 mmHg or diastolic blood pressure ≥ 90 mmHg from a single blood pressure reading taken during a participant's clinic visit at the primary healthcare clinic. Elevated blood pressure was dichotomized into a “yes/no” binary variable. All the participants with normal blood pressure: systolic blood pressure: 120–130 mmHg or diastolic blood pressure 80–85 mmHg and high-normal blood pressure: systolic blood pressure 130–140 mmHg or 85–90 mmHg were grouped as “no” for blood pressure that is not elevated.

### 2.6 Data analysis

Frequencies were calculated to describe categorical variables while median and interquartile range were calculated for continuous variables. The univariate analysis assessed the associations between outcomes (elevated blood pressure) and covariates and unadjusted odds ratios (OR) and their corresponding 95 % confidence intervals (95 % CI) were reported. Variables that were associated with the outcome (elevated blood pressure) with a significance level of <0.05 were subsequently included in a multivariable logistic regression analysis to assess independent associations; *p* < 0.05 was considered statistically significant. Stratified analyses of substance use (by “alcohol”, “tobacco” and “alcohol and tobacco”) were performed. Adjusted odds ratios (aOR) and their corresponding 95 % confidence intervals (95 % CI) were reported. Data was analysed using STATA version 17.

## 3 Results

### 3.1 Participant's characteristics

Participants' demographic characteristics are presented in Table 1. Of the 2148 participants included, 57.3 % (1230/2148) were males. The participants' median age was 41 years (IQR 35–48 years). Almost two-thirds of the participants (64.8 %, 1392/2148) self-reported drinking alcohol only while one in five (22.9 %, 491/2148) reported usage of alcohol and tobacco. Most of the participants (81.1 %, 1741/2148) were living with HIV and 10.5 %, (226/2148) participants had comorbidities. One in four participants (18.7 %, 402/2148) were diagnosed with hypertension (two consecutive elevated blood pressure readings) and 16.9 %, (364/2148) were on anti-hypertensive treatment. Two-thirds of people living with HIV were on antiretroviral treatment (66.2 %, 1153/1741).

**Table 1:** Participants' characteristics categorized by blood pressure status for participants who self-reported substance use in the Integrating HIV and Heart Health in South Africa intervention study between August 2022 and May 2024.

	**Total**	**Elevated blood pressure**	**High-normal**	**Normal**
**Median age (IQR) in years**	41 (35–48)	44 (38–52)	41 (31–47)	38 (33–45)
	**Total (n, %)**	**Elevated blood pressure** **(n, %)**	**High-normal (n, %)**	**Normal (n, %)**
**Age category (n = 2148)**				
18–29 years	199 (9.3)	35 (4.5)	30 (6.5)	134 (14.7)
30–39 years	734 (34.2)	197 (25.5)	170 (36.8)	367 (40.2)
40–49 years	744 (34.6)	282 (36.5)	180 (39.0)	282 (30.9)
50+ years	471 (21.9)	258 (33.4)	82 (17.8)	131 (14.3)
**Sex (n = 2148)**				
Female	918 (42.7)	279 (36.1)	185 (40.0)	454 (49.7)
Male	1230 (57.3)	493 (63.9)	277 (60.0)	460 (50.3)
**Substance use (n = 2148)**				
Alcohol only	1392 (64.8)	494 (64.0)	311 (67.3)	587 (64.2)
Tobacco only	246 (11.5)	105 (13.6)	41 (8.9)	100 (10.9)
Tobacco and Alcohol	491 (22.9)	168 (21.8)	105 (22.7)	218 (23.9)
Alcohol, Tobacco and other drugs	19 (0.9)	5 (0.7)	5 (1.1)	9 (1.0)
**HIV status (n = 2148)**				
HIV negative	351 (16.3)	216 (28.0)	52 (11.3)	83 (9.1)
HIV positive	1741 (81.1)	527 (68.3)	397 (85.9)	817 (89.4)
HIV unknown	56 (2.6)	29 (3.8)	13 (2.8)	14 (1.5)
**Comorbidities (*n* = 1236)**				
Yes	226 (18.3)	136 (33.7)	47 (17.0)	43 (7.8)
No	1010 (81.7)	268 (66.3)	230 (83.0)	512 (92.3)
**Hypertension treatment (*n* = 1464)**				
Yes	364 (24.9)	226 (42.6)	69 (21.9)	69 (11.2)
No	1100 (75.1)	304 (57.4)	246 (78.1)	546 (88.8)
**Antiretroviral treatment (*n* = 1741)**				
Yes	1153 (66.2)	354 (67.2)	264 (66.5)	535 (46.9)
No	588 (33.8)	173 (32.8)	133 (33.5)	282 (34.5)

Descriptions: Normal blood pressure, systolic blood pressure 120–130 mmHg or diastolic blood pressure 80–85 mmHg; High-normal blood pressure, systolic blood pressure 130–140 mmHg or 85–90 mmHg; Elevated blood pressure, systolic blood pressure of ≥ 140 mmHg or diastolic blood pressure of ≥ 90 mmHg.

Abbreviations: IQR, interquartile range; n, number; %, percentage.

### 3.2 Study outcome

Of all study participants with measured blood pressure (single reading), 42.6 % (914/2148) of those had normal blood pressure, 21.5 % (462/2148) had high-normal blood pressure and 35.9 % (772/2148) had elevated blood pressure.

### 3.3 Univariate analysis

Results of univariate analyses of correlates of elevated blood pressure are shown in Table 2. The risk factors for elevated blood pressure were participants who are 30–39 years old (OR 1.71 [95 %CI 1.15–2.55], 40–49 years old (OR 2.84 [95 %CI 1.92–4.22]), and 50+ years (OR 5.62 [95 %CI 3.74–8.45]), males (OR 1.53 [95 %CI 1.27–1.83], smoking tobacco (OR 1.37 [95 %CI 1.04–1.80], HIV negative (OR 3.66 [95 %CI 2.88–4.64] and unknown status (OR 2.38 [95 %CI 1.39–4.08], having comorbidities (OR 4.11 [95 %CI 3.04–5.55]) and being on hypertension treatment (OR 4.21 [95 %CI 3.28–5.41].

**Table 2:** Univariable analyses of factors associated with elevated blood pressure among participants who self-reported substance usage in the Integrating HIV and Heart Health in South Africa intervention study between August 2022 and May 2024.

Variables	Crude/unadjusted odds ratio	[95 % confidence interval]
Age		
18–29 years	Ref	
30–39 years	1.71	1.15–2.55
40–49 years	2.84	1.92–4.22
50+	5.62	3.74–8.45
Sex		
Female	Ref	
Male	1.53	1.27–1.83
Substance use		
Alcohol only	Ref	
Tobacco only	1.37	1.04–1.80
Tobacco and alcohol	0.95	0.77–1.18
Tobacco, alcohol, and other drugs	0.65	0.23–1.82
HIV status		
HIV positive	Ref	
HIV negative	3.66	2.88–4.64
HIV unknown status	2.38	1.39–4.08
**Comorbidities**		
No	Ref	
Yes	4.11	3.04–5.55
Hypertension treatment		
No	Ref	
Yes	4.21	3.28–5.41
Antiretroviral treatment		
No	Ref	
Yes	1.07	0.86–1.33

Abbreviations: Ref, reference.

### 3.4 Multivariable logistic regression analysis

Results of multivariable logistic regression analyses of correlates of elevated blood pressure are shown in Table 3. The independent risk factors for elevated blood pressure were participants who are 30–39 years old (aOR 2.78 [95 %CI 1.42–5.41], 40–49 years old (aOR 3.81 [95 %CI 1.95–7.46]), and 50+ years (aOR 5.00 [95 %CI 2.45–10.20]) and having comorbidities (aOR 2.35 [95 %CI 1.31–4.22]).

**Table 3:** Multivariable logistic regression of factors associated with elevated blood pressure among participants who self-reported substance usage in the Integrating HIV and Heart Health in South Africa intervention study between August 2022 and May 2024.

Variables	Adjusted odds ratio	[95 % Confidence interval]
Age		
18–29 years	Ref	
30–39 years	2.78	1.42–5.41
40–49 years	3.81	1.95–7.46
50+	5.00	2.45–10.20
Substance use		
Alcohol only	Ref	
Tobacco only	0.80	0.51–1.25
Tobacco and alcohol	0.84	0.61–1.16
Tobacco, alcohol, and other drugs	1.63	0.40–6.74
Sex		
Female	Ref	
Male	1.17	0.88–1.55
HIV status		
HIV positive	Ref	
HIV negative	0.90	0.48–1.70
HIV unknown status	0.85	0.29–2.47
Comorbidities		
No	Ref	
Yes	2.35	1.31–4.22
Hypertension treatment		
No	Ref	
Yes	1.58	0.84–2.98
Antiretroviral treatment		
No	Ref	
Yes	0.66	0.14–3.21

Notes: Analysis adjusted for age, substance use, sex, HIV status, comorbidities, hypertension treatment and antiretroviral treatment.

**Abbreviations:** Ref, reference.

When stratified by alcohol use (Table 4), independent risk factors for elevated blood pressure remained unchanged, that participants aged 30–39 years old (aOR 3.18 [95 %CI 1.40–7.24]), 40–49 years old (aOR 4.56 [95 %CI 1.99–10.49]), 50+ years old (aOR 5.79 [95 %CI 2.36–14.23]), and those who have comorbidities (aOR 2.21 [95 %CI 1.07–4.54]) were independent risk factors for elevated blood pressure. No associations between elevated blood pressure and covariates were identified when the analyses were stratified by tobacco.

**Table 4:** Multivariable logistic regression of factors associated with elevated blood pressure among participants who self-reported substance usage (alcohol) in the Integrating HIV and Heart Health in South Africa project between August 2022 and May 2024.

Variables	Adjusted odds ratio	[95 % confidence interval]
Age		
18–29 years	Ref	
30–39 years	3.18	1.40–7.24
40–49 years	4.56	1.99–10.49
50+ years	5.79	2.36–14.23
Sex		
Female	Ref	
Male	1.14	0.81–1.60
HIV status		
HIV positive	Ref	
HIV negative	1.46	0.59–3.59
HIV unknown status	2.29	0.40–13.03
Comorbidities		
No	Ref	
Yes	2.21	1.07–4.54
Hypertension treatment		
No	Ref	
Yes	1.62	0.73–3.59
Antiretroviral treatment		
No	Ref	
Yes	2.20	0.20–24.16

Note: Analysis adjusted for age, substance use, sex, HIV status, comorbidities, hypertension treatment, and antiretroviral treatment.

Abbreviations: IQR: Interquartile Range; Ref, reference.

## 4 Discussion

To our knowledge this is the first report of substance use and blood pressure in the region. This study reported on the prevalence of elevated blood pressure and examined factors associated with elevated blood pressure among substance users in selected primary healthcare clinics in an urban part of Johannesburg, South Africa. Chronic use of substances like alcohol, tobacco, methamphetamine, and cocaine has been linked to elevated blood pressure levels (American Heart Association, 2024; Aronow, 2017). Participants self-reported their substance use, including alcohol and tobacco. Prevalence of elevated blood pressure among the substance users were as follows: 64 % for those who took only alcohol, 13.6 % for those who took only tobacco, 21.8 % for those who took both tobacco and alcohol, and 0.7 % for those who took alcohol, tobacco, and other drugs like dagga (Table 1).

From the univariate analysis (Table 2), examining each variable in isolation, older age, male sex at birth, self-reported tobacco use, HIV-negative or unknown HIV status, presence of comorbidities, and receiving hypertensive treatment were all significantly associated with elevated blood pressure. These findings on the relationship between these factors and elevated blood pressure are critical because existing literature shows that it can induce the development of cardiovascular disease (Fuchs and Whelton, 2020). A plausible explanation for these significant factors associated with elevated blood pressure (which is a risk factor for cardiovascular disease) is that older age and male sex are well-established risk factors for high blood pressure, owing to age-related vascular changes and potential hormonal influences (Whelton et al., 2018; Connelly et al., 2022). Further evidence suggests that men have an increased risk of elevated blood pressure and developments of cardiovascular diseases like hypertension compared to women because of p socioeconomic inequalities (Minzer et al., 2020; Di Federico et al., 2023; Algharably et al., 2024; Connelly et al., 2022). Further, as a potent vasoconstrictor, nicotine in tobacco directly contributes to elevated blood pressure (Oparil et al., 2018; Sanjuan et al., 2014; American Heart Association, 2024).

Moreover, the significant relationship between HIV negative status and increased odds of elevated blood pressure in the univariate analysis aligns with findings where HIV-negative people have been shown to have more traditional cardiovascular risk factors (Masyuko et al., 2020). Although there was no significant association found in the univariate analysis, it is important to note that factors like antiretroviral treatment and inflammations caused by HIV-related conditions can influence the complex relationship between an HIV positive status and elevated blood pressure, which can lead to cardiovascular health concerns (So-Armah et al., 2020). Additionally, the associations identified between comorbidities and elevated blood pressure is unsurprising, as they can induce inflammation and vascular damage, thereby heightening the risk of hypertension and other cardiovascular diseases. While it may appear paradoxical, the relationship identified between hypertensive treatment and elevated blood pressure could probably be because people with severe or uncontrolled hypertension are more likely to be prescribed medication.

Nonetheless, the multivariate analysis (Table 3), after adjusting for potential confounding variables, indicated that only age and the presence of comorbid conditions showed a significant association with elevated blood pressure. This indicates that although other factors exhibited individual associations, their influence on blood pressure may be mediated or confounded by these two fundamental factors– age and having comorbid conditions. Increasing age that emerged as an independent predictor was such that those in the 50 years and above age bracket had greater odds of elevated blood pressure than those in the youngest age bracket, from to the multivariate analysis. This finding aligns with previous studies that found that the cardiovascular system changes physiologically with age, making ageing a significant risk factor (Zhao et al., 2023; Okojie et al., 2020). Also, the presence of comorbidities was consistently seen to be associated with an increased likelihood of elevated blood pressure in both the univariate and multivariate analysis highlighting the interconnected nature of various health conditions and underscores the importance of adopting a holistic approach to healthcare. The relationship between comorbidities and elevated blood pressure is multifaceted as many comorbidities, such as obesity, diabetes, and chronic kidney disease, share common risk factors with elevated blood pressure, including genetic predisposition, inflammatory oxidative stress, unhealthy lifestyle choices (poor diet, physical inactivity), and socioeconomic factors (Whelton et al., 2018; Ojangba et al., 2023). Additionally, evidence states that substance use can worsen the management and progression of chronic diseases like diabetes and kidney disease, further increasing the risk of elevated blood pressure and cardiovascular events (Whelton et al., 2018).

### 4.1 Limitations

The study's limitations, such as use of secondary data, cross-sectional design, exclusion of data collected at follow-up visits, and reliance on self-reported data on substance use, make it difficult to draw definitive conclusions regarding the relationship between cause and effect. The analysis was limited to variables available in the secondary data, so potential confounding variables like participants' dietary habits, level of physical activity, and preexisting medical conditions could not be included. In addition, exploring specific types of comorbidities and their contributions to blood pressure elevation would also provide valuable insights.

## 5 Conclusion

This study utilized secondary analysis of the Integrating HIV and Heart Health in South Africa intervention study data to explore trends in elevated blood pressure and associated factors among individuals receiving routine clinical care for chronic conditions, like HIV, who self-reported substance use. The findings highlight an increased prevalence of elevated blood pressure among individuals reporting substance use at selected primary healthcare clinics in urban Johannesburg, South Africa. Age and comorbidities emerged as significant risk factors for elevated blood pressure, but the precise interplay between these factors and substance use patterns remains unclear and warrants further investigation. While age and comorbidities are established risk factors for hypertension, the complex interactions with substance use are poorly understood, particularly in sub-Saharan Africa, where demographic shifts and evolving substance use patterns may influence hypertension prevalence. Previous research (Sanchez-Roige et al., 2022; Yoon et al., 2020) underscores the importance of further studies to elucidate the shared pathways linking substance use, age, and comorbid conditions, potentially leading to more personalized approaches to hypertension management. To establish causal relationships, future research should prioritize prospective cohort study designs in South Africa and across the African continent. These studies should incorporate diverse countries, such as South Africa, Nigeria, Egypt, Ethiopia, and Congo, to account for varying lifestyles, cultural contexts, and dietary patterns that may confound findings. Lastly, it is crucial to implement public health initiatives that address the long-term health effects of substance use, with a focus on its interaction with age and pre-existing health conditions, to mitigate risks and improve outcomes for vulnerable populations.

## Funding/support

Research reported in this publication is supported by the National Heart, Lung, And Blood Institute of the National Institutes of Health under Award Number UH3HL156388 and Fogarty International Center. The content is solely the responsibility of the authors and does not necessarily represent the official views of the National Institutes of Health.

## Statement of human and animal rights and informed consent

This study involved the use of secondary data from the Integrating HIV and Heart Health in South Africa intervention study. Ethical approvals and necessary permissions to access patients' medical records s was obtained from the University of the Witwatersrand Institutional Review Board, South Africa and the Johannesburg District Research Committee.

## Authors disclosures

All authors declare that there are no financial and personal relationships with other people or organizations that could inappropriately affect this study and publication.

## CRediT authorship contribution statement

**K.E. Oladimeji:** Writing – review & editing, Writing – original draft, Visualization, Validation, Methodology, Conceptualization. **S. Gumede:** Writing – review & editing, Writing – original draft, Visualization, Validation, Project administration, Methodology, Investigation, Formal analysis, Data curation. **A. Nyatela:** Writing – review & editing, Writing – original draft, Project administration, Methodology, Investigation, Data curation, Conceptualization. **S. Nonyukela:** Writing – review & editing, Methodology, Investigation, Data curation. **R. Mohale:** Writing – review & editing, Project administration, Methodology, Investigation, Data curation. **S.T. Lalla-Edward:** Writing – review & editing, Writing – original draft, Visualization, Validation, Supervision, Software, Resources, Project administration, Methodology, Investigation, Funding acquisition, Formal analysis, Data curation, Conceptualization. **D. Dwarka:** Writing – review & editing, Writing – original draft, Visualization, Validation, Project administration, Methodology, Investigation, Formal analysis, Data curation.

## Declaration of competing interest

The authors declare that they have no known competing financial interests or personal relationships that could have appeared to influence the work reported in this paper.

## Data Availability

Data will be made available on request.
